# Overcoming Antigenic Drift in PEDV: Broadly Protective Antigen Design and sIgA-Driven Lactogenic Immunity

**DOI:** 10.3390/vetsci13070647

**Published:** 2026-06-30

**Authors:** Qiao-Qiao Zhang, Hao-Jie Zhang, Lan-Lan Zheng, Yue Zhang, Hong-Ying Chen, Shi-Jie Ma

**Affiliations:** 1 College of Veterinary Medicine, Henan Agricultural University, Zhengdong New District Longzi Lake 15#, Zhengzhou 450046, China; qiaoqiaozhang2026@163.com (Q.-Q.Z.); zzhjj20@163.com (H.-J.Z.); lanlan@henau.edu.cn (L.-L.Z.); 2 Ministry of Education Key Laboratory for Animal Pathogens and Biosafety, Zhengzhou 450046, China; 3 Henan Province Key Laboratory for Animal Food Pathogens Surveillance, Zhengzhou 450046, China

**Keywords:** porcine epidemic diarrhea virus, maternal mucosal immunity, secretory IgA, gut–mammary gland–sIgA axis

## Abstract

Porcine epidemic diarrhea virus (PEDV) remains one of the most critical enteric coronaviruses affecting the global swine industry. PEDV causes severe diarrhea, dehydration, and high mortality in neonatal piglets. Despite the widespread use of commercial vaccines, persistent PEDV outbreaks worldwide indicate that current vaccination strategies provide suboptimal protection. Increasing evidence suggests that the limited effectiveness of current control strategies is primarily attributable to two interrelated factors: continuous antigenic variation among circulating PEDV strains and inadequate induction of maternal mucosal immunity. Unlike systemic viral infections, effective protection against PEDV in neonatal piglets predominantly depends on lactational immunity mediated by the gut–mammary gland–secretory IgA (sIgA) axis. However, most currently available vaccines predominantly induce systemic IgG responses and fail to effectively stimulate intestinal immune imprinting or sustain sIgA secretion in colostrum and milk. In this review, we summarize the mechanisms underlying PEDV evolution and maternal mucosal immunity, with particular emphasis on the gut–mammary gland–sIgA axis. We further discuss the recent advances in and limitations of current vaccine platforms and propose an integrated framework for broadly protective PEDV vaccines based on structural antigen optimization, mucosal-targeted immunization, and sIgA-oriented evaluation systems. This framework may provide new insights into the rational design of more effective maternal vaccines against PEDV and other enteric coronaviruses affecting pigs.

## Simple Summary

Porcine epidemic diarrhea virus (PEDV) continues to impose a significant burden on the swine industry, particularly because neonatal piglets depend on maternally acquired immunity for protection during the first weeks of life. Despite the widespread use of commercial vaccines, PEDV is difficult to control owing to the continuous emergence of antigenically distinct variants and the insufficient ability of current vaccines to induce effective mucosal immunity in pregnant sows. Protection of suckling piglets mainly depends on secretory IgA (sIgA) antibodies delivered through colostrum and milk, underling the central role of lactational immunity in disease prevention. This review summarizes PEDV evolution, maternal mucosal immune responses, and recent advances in vaccine development. It also discusses the challenges facing existing vaccine strategies and highlights potential approaches to improve future maternal vaccines though optimized antigen design, enhanced mucosal immunization, and more comprehensive evaluation of protective immunity.

## 1. Introduction

Since its initial identification in Europe during the 1970s and 1980s, porcine epidemic diarrhea (PED) has remained prevalent in major swine-producing regions worldwide. PED is an acute and highly contagious enteric disease characterized by diarrhea, vomiting, and dehydration, with particularly high mortality rates in neonatal piglets [1,2]. In the absence of adequate lactogenic immunity, mortality rates in neonatal piglets may approach 100%, highlighting the critical role of maternally derived immune protection [3]. The etiological agent, porcine epidemic diarrhea virus (PEDV), primarily infects villous epithelial cells of the small intestine, resulting in villous atrophy, disruption of intestinal tight junctions, reduced mucus secretion, and decreased digestive enzyme activity. These pathological changes ultimately lead to malabsorption and impaired digestive function [4]. PEDV is primarily transmitted via the fecal–oral route through direct contact with infected feces or vomitus. Fomites, including contaminated feed, vehicles, equipment, and environmental surfaces, contribute to viral dissemination both within and between farms [5,6]. In addition, PEDV has been shown to spread via airborne aerosols, and virus-bearing CD3+ T cells may facilitate viral dissemination from the nasal mucosa to the intestinal tract through the circulatory system, representing an alternative route of enteric infection [7].

Although inactivated and live attenuated vaccines are widely used, current vaccination strategies provide inconsistent protection and frequently fail to prevent viral shedding under field conditions [8,9]. One major limitation is that most conventional vaccines are administered intramuscularly and predominantly induce systemic IgG response, which is insufficient to fully provide complete protection at the intestinal mucosa, the primary site of PEDV infection [10,11].

For the enteric coronavirus PEDV, effective protection depends primarily on intestinal mucosal immunity rather than systemic antibody responses [12,13]. Among mucosal immune effectors, secretory IgA (sIgA) plays a central role in neutralizing viral particles within the intestinal lumen and preventing infection of intestinal epithelial cells [13,14]. Several studies in swine have demonstrated that sIgA levels in colostrum and milk are more strongly associated with protection in neonatal piglets than serum neutralizing antibody titers, highlighting the critical role of mucosal immunity in protection against PEDV [15,16]. Therefore, maternally derived lactogenic immunity is considered the primary natural defense against PEDV infection in suckling piglets [17,18]. Because swine possess an epitheliochorial placenta, piglets are born with little or no circulating immunoglobulins and early protection relies on antibodies acquired through colostrum and milk [13,17]. This process is mediated by the migration of IgA-secreting plasmablasts from gut-associated lymphoid tissue (GALT) to the mammary gland, thereby establishing the gut–mammary gland–sIgA axis [19].

This review systemically summarizes recent advances in PEDV vaccine research, with particular emphasis on maternal mucosal immunity and sIgA-based evaluation strategies. Additionally, we discuss strategies for broad-spectrum antigen design and structural optimization to facilitate development of next-generation vaccines. Collectively, these advances provide new insights into the rational design and effective control of PEDV.

## 2. Etiology and Prevalence of PEDV

### 2.1. The PEDV Genome and S Protein

PEDV is an enveloped, positive-sense, single-stranded RNA virus belonging to the genus *Alphacoronavirus* within the family *Coronaviridae* and the order *Nidovirales* [20,21]. PEDV virions are 95–190 nm in diameter and possess surface projections measuring about 18 nm [22,23]. The PEDV genome is approximately 28 kb in length, containing a 5′ cap, a 3′ poly (A) tail, and seven open reading frames (ORFs). These ORFs encode four structural proteins, namely spike (S), membrane (M), envelope (E), and nucleocapsid (N), as well as the accessory protein ORF3 and 16 nonstructural proteins (nsp) [24]. Among these proteins, the S protein comprising 1383 amino acids (aa) is a type I transmembrane glycoprotein located on the viral surface and is considered the major protective antigen of PEDV. It mediates viral attachment, receptor binding, and membrane fusion, and therefore plays critical roles in viral infectivity, tissue tropism, and host immune responses [25]. Structurally, the PEDV S protein exists as a prefusion trimer composed of the S1 and S2 subunits. The S1 subunit is primarily responsible for receptor binding, whereas the S2 subunit mediates membrane fusion. The S1 subunit contains the signal peptide (SP, aa1–18), the N-terminal domain (NTD), and the C-terminal receptor-binding domain (CTD/RBD). This subunit contains several major neutralizing epitopes and represents the principal region undergoing antigenic variation. In contrast, the S2 subunit is relatively conserved and mainly contains the fusion peptide (FP, aa891–908), heptad repeat region 1 (HR1, aa978–1117), heptad repeat region 2 (HR2, aa1274–1313), the transmembrane domain (TM, aa1328–1350), and the cytoplasmic tail (CT, aa1351–1386) [26,27].

**Figure 1:**
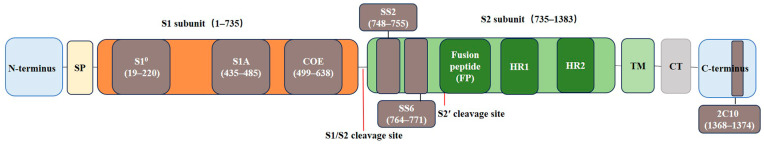
Schematic diagram of the S protein and major neutralizing epitopes. The S protein is composed of two functional subunits: the receptor-binding S1 subunit (residues 1–735) and the membrane-fusion S2 subunit (residues 735–1383). The S1 subunit contains the signal peptide (SP), the N-terminal domain (NTD), and several major neutralizing epitopes, including S1^0^, S1A, and COE. The S2 subunit contains the fusion peptide (FP), heptad repeat domains 1 and 2 (HR1 and HR2), the transmembrane domain (TM), and the cytoplasmic tail (CT). Three major neutralizing epitopes are highlighted, including SS2 (amino acids 748–755), SS6 (amino acids 764–771) and 2C10 (amino acids 1368–1374). S1/S2 and S2′ are also indicated. Different colors indicate distinct structural regions of the PEDV S protein, whereas the gray boxes represent major antigenic epitopes or functional domains.

To date, multiple neutralizing antigenic epitopes have been identified within the PEDV S protein, including S1^0^, S1A, COE, SS2, SS6, and 2C10 [28,29,30]. Among these epitopes, COE is located in the C-terminal region of the S1 subunit and is one of the most extensively characterized neutralizing epitopes in PEDV (Figure 1). In contrast, SS2, SS6, and 2C10 are located within the S2 subunit and are highly conserved, making them attractive targets for the development of broadly protective vaccine antigens [28,29,30].

### 2.2. Pathogenesis and Clinical Manifestations

PEDV primarily infects intestinal villous epithelial cells in pigs, causing severe watery diarrhea and high mortality through a pathogenic process involving viral invasion, immune evasion, cellular injury, and intestinal barrier disruption [31]. PEDV initiates infection through interactions between S protein and host-cell surface factors followed by extensive viral replication in the jejunum and ileum. A hallmark of PEDV pathogenesis is its ability to evade and suppress host innate immunity responses. Multiple PEDV proteins, including nsp1, nsp3, nsp5, nsp15, and N protein, target pattern recognition receptor (PRR)-associated signaling pathways. Specifically, PEDV interferes with viral RNA recognition and suppresses the RIG-I/MDA5-MAVS-TBK1-IRF3 signaling cascade. Consequently, the production of type I interferons (IFN-α/β), type III interferons (IFN-λ), and interferon-stimulated genes (ISGs) is markedly reduced. Impairment of these antiviral responses facilitates viral replication within intestinal epithelial cells [32,33,34].

**Figure 2:**
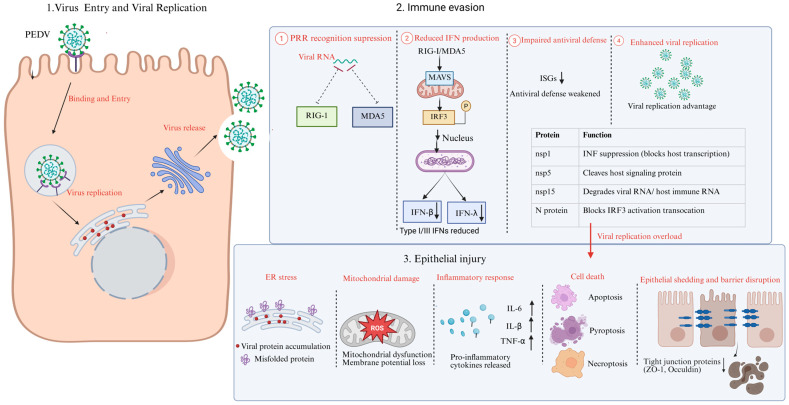
Schematic illustration of the pathogenesis of PEDV. PEDV invades and infects intestinal villus epithelial cells, where it establishes efficient intracellular replication. During infection, PEDV impairs the recognition function of pattern recognition receptors (PRRs) and suppresses the production of type I and type III interferons, thereby inhibiting innate antiviral immune responses, facilitating viral replication, and promoting immune evasion. In addition, PEDV induces epithelial cell death and disrupts intestinal barrier integrity. Dashed arrows indicate the antagonistic effects of PEDV on host antiviral signaling mediated by RIG-I and MDA5.

In addition to immune evasion, PEDV infection induces profound cytopathic effects. PEDV infection induces mitochondrial enlargement, endoplasmic reticulum (ER) stress, and nuclear membrane damage, which collectively disrupt cellular homeostasis and activate multiple programmed cell death pathways. Progressive loss of villous epithelial cells subsequently results in villous atrophy, decreased expression of tight junction proteins, impaired intestinal barrier integrity, and defective absorption of water and electrolytes. Collectively, these pathological alterations underlie the clinical manifestations of PEDV infection, including severe watery diarrhea, dehydration and significant mortality in neonatal piglets (Figure 2).

### 2.3. Global Epidemiology and Prevalence

PEDV was first reported in the United Kingdom in 1971 and was officially recognized as the causative agent of porcine epidemic diarrhea in 1978 [35]. Over the following three decades, PEDV circulated predominantly in sporadic and localized outbreaks across Europe and Asia. However, since 2010, large-scale PED outbreaks caused by highly pathogenic PEDV variants have occurred in China, resulting in mortality rates of up to 80–100% in neonatal piglets, marking a new phase in the epidemiology of the disease [36,37].

**Figure 3:**
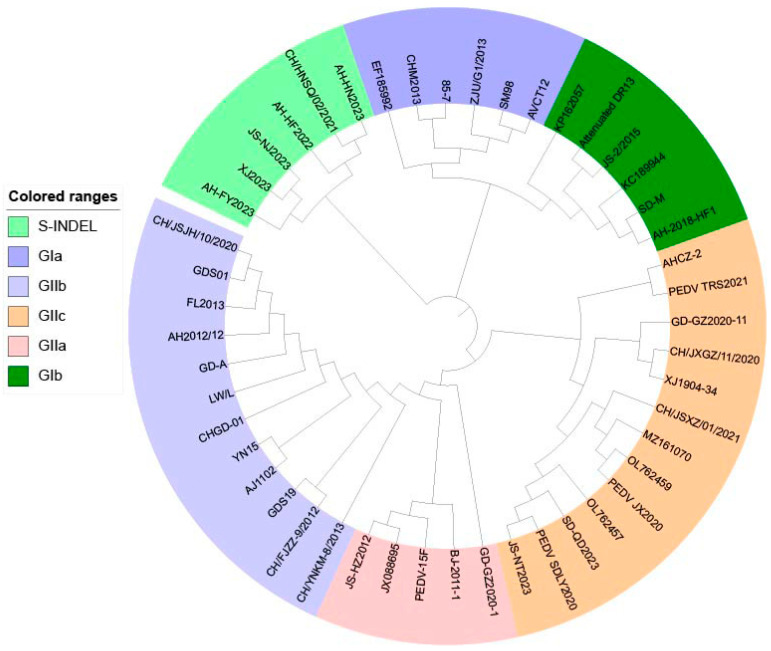
**Phylogenetic analysis of PEDV S gene** [45]. A phylogenetic tree was constructed using the maximum likelihood method in MEGA 12.0 software based on 48 published PEDV S gene sequences, with bootstrap values calculated from 1000 replicates.

Based on the whole-genome and S gene sequences, PEDV strains can be broadly classified into two major groups, G1 and G2. Phylogenetic analyses revealed that the circulating PEDV strains differed significantly from the classical CV777 strain and progressively evolved into variant lineages represented by the G2 group. Among these lineages, G2a and subsequently emerging G2b strains became predominant in China and other Asian regions [38]. In 2013, PED outbreaks were first reported in the United States and spread rapidly to multiple swine-producing states across North America within a few months. These outbreaks marked the global establishment of highly pathogenic PEDV G2 variants [39,40]. Specifically, the G2c subgroup has rapidly disseminated across China from late 2024, progressively replacing dominant G2a/G2b lineages. Surveillance studies conducted in various Chinese provinces between 2024 and 2025 have confirmed this significant upward trend, suggesting ongoing lineage replacement [41,42,43]. This shift is thought to be driven, at least in part, by mutations in the S protein, as critical neutralizing epitopes exhibit substantial sequence variability and altered glycosylation patterns, potentially enhancing immune evasion [41,44]. Circulating PEDV strains have diverged substantially from classic strains, and are characterized by the continuous evolution and turnover of G2 variants (Figure 3).

## 3. Evolutionary Challenges in PEDV Prevention and Control

The genetic diversity of the S protein is a major driving force underlying PEDV evolution. The S protein mediates receptor recognition, membrane fusion, and viral entry, and serves as the principal target of neutralizing antibodies, thereby being subjected to strong host immune pressure [1,25,46]. Consequently, most phylogenetic classification systems, including the widely used G1 and G2 group classification, are based primarily on S gene diversity rather than variation in other genomic regions [46]. Continuous mutations, insertions/deletions, and changes in glycosylation patterns within the S1 subunit are considered major contributors to antigenic drift and reduced vaccine efficacy [29]. Interestingly, amino acid substitutions are enriched within key neutralizing epitopes, including the S-NTD, COE, SS2, SS6 and 2C10 areas.

Although the S protein is the primary determinant of antigenic evolution, several non-spike proteins also contribute to viral adaptation, virulence, and immune evasion. For example, the accessory protein ORF3 functions as an important virulence factor by modulating ion channel activity, viral replication efficiency, cell tropism, and host immune regulation [47]. In addition, several nonstructural proteins, particularly nsp1, nsp3, nsp5, nsp14, nsp15 and nsp16, antagonize interferon signaling pathways and suppress innate immunity responses, thereby promoting viral persistence under host immune pressure [34,48]. Nevertheless, these proteins are relatively conserved compared with the highly variable S protein and mainly contribute to maintaining viral fitness rather than altering its antigenic properties [49].

Recent comparative analyses have shown that the currently dominant G2c lineage has undergone continuous and extensive amino acid diversification within these antigenic regions. Common substitutions within the S-NTD area include S27L, E57A, N139D, M214T, and P229L. Moreover, numerous amino acid changes have been identified within the COE neutralizing epitope (aa 499–638), especially at residues proximal to the receptor-binding region, including A520S/L, F539L, K566N, D569E, G612V, F615L, P634S, and E636V/K, many of which occur at relatively high frequencies. Furthermore, residue Y1376 within the 2C10 epitope appears to be particularly prone to mutate [41,42,50]. These alterations may affect the epitope conformation and antibody accessibility, thereby reducing the neutralization sensitivity and facilitating antigenic drift under vaccine-induced immune pressure. Besides the sequence mutation, dynamic changes in S protein N-glycosylation also contribute to the antigenic evolution of PEDV. Longitudinal analyses of circulating strains indicate that recent G2c strain isolates tend to lose N510 and N347 glycosylation sites, while occasionally acquiring new sites at N340 and N127. These changes may mask critical neutralizing epitopes through glycan shielding, thereby altering viral antigenicity and its sensitivity to antibody-mediated neutralization [41,42].

Evidence from reverse genetics studies further supports the role of glycan shielding in PEDV antigenic evolution. Recombinant PEDV lacking glycosylation sites within the receptor-binding domain (RBD) retained its pathogenicity but elicited significantly higher IgG and neutralizing antibody responses in infected animals [51]. These findings suggest that glycosylation within the RBD may function as a physical barrier that impedes antibody recognition and binding.

In addition to immune evasion, mutation analyses of N-glycosylation sites have demonstrated their essential role in the viral life cycle. Site-directed mutagenesis of several predicted N-glycosylation sites revealed that disrupting N118, N216, N726, N1232 or N1249 significantly affects viral replication and decreases plaque size [52]. These glycosylation sites are therefore crucial for proper S protein folding and for maintaining efficient viral replication and infectivity. N-glycosylation regulates S protein maturation, viral entry, host interactions, and adaptation to host environments [52,53,54].

In contrast to the highly variable S1 subunit, the S2 subunit is relatively conserved. Key functional components of the protein, including FP, HR and TM, remain almost unchanged across different strains [55]. These regions are critical for membrane fusion, and their amino acid sequences exhibit minimal variation during antigenic drift because of strong structural and functional constraints.

In summary, the molecular basis of PEDV antigen variation involves three major features: (i) extensive sequence variability within the S1 subunit, including amino acid substitutions in neutralizing epitopes such as COE and SS6 [56]; (ii) the loss, shift or acquisition of N-glycosylation sites that modulate epitope accessibility and antibody recognition [52,57]; and (iii) the highly conserved S2 subunit, which is maintained by strong structural and functional constraints [29]. Together, these features create an evolutionary architecture characterized by a highly variable S1 immune-recognition region and a conserved S2 functional core.

Collectively, these findings suggest that antigenic evolution of the PEDV S protein has progressively reduced the antigenic match between classical vaccine strains and contemporary circulating variants. This evolutionary trend may partially account for the limited cross-protective efficacy of conventional PEDV vaccines under field conditions.

## 4. The Immunological Basis of PEDV Protection: Maternal sIgA

PEDV primarily infects mature intestinal epithelial cells in the small intestine and causes severe diarrhea, dehydration, and high mortality in neonatal piglets. Therefore, effective immune protection against PEDV requires not only the induction of antibody responses but also the establishment of a sustained local immune barrier at the primary site of viral invasion, namely the intestinal mucosal surface. Unlike viruses that mainly rely on systemic IgG-mediated protection, PEDV, as a typical enteric coronavirus, primarily depends on sIgA immunity in the intestinal mucosa. The sIgA can neutralize viral particles at the intestinal surface, block viral attachment, and reduce viral replication in intestinal epithelial cells. Consequently, the continuous intake of maternal mucosal antibodies during the lactation period is critical for protecting neonatal piglets against PEDV infection [13,58,59].

### 4.1. Lactogenic Immunity

However, neonatal piglets cannot rapidly develop effective adaptive immunity. PEDV infection usually occurs within the first few days after birth, when the immune system is still immature. The establishment of humoral immunity, including B-cell activation and plasma cell differentiation, typically takes 2–3 weeks. In addition, maternal antibodies, especially milk-derived sIgA and IgG, can neutralize vaccine strains and suppress the replication of live attenuated vaccines in the intestine, thereby interfering with the induction of acquired immunity in piglets [13]. Currently, PEDV prevention and control strategies mainly focus on vaccinating pregnant sows to enhance maternal milk antibody levels and provide passive mucosal protection for piglets during the lactation period.

This dependence on maternal antibodies is closely associated with the unique placental structure of swine. Pigs possess an epitheliochorial placenta in which maternal and fetal circulations are separated by multiple layers of intact tissue, thereby greatly limiting transplacental transfer of maternal immunoglobulins. Consequently, piglets are born in a state of agammaglobulinemia or severe hypogammaglobulinemia and therefore lack sufficient passive immune protection at birth. Early postnatal immunity in piglets primarily depends on the ingestion of colostrum and milk containing maternal antibodies. Colostrum mainly provides systemic IgG, whereas sIgA in mature milk continuously mediates local intestinal mucosal protection [60,61].

### 4.2. The Gut–Mammary Gland–sIgA Axis

**Figure 4:**
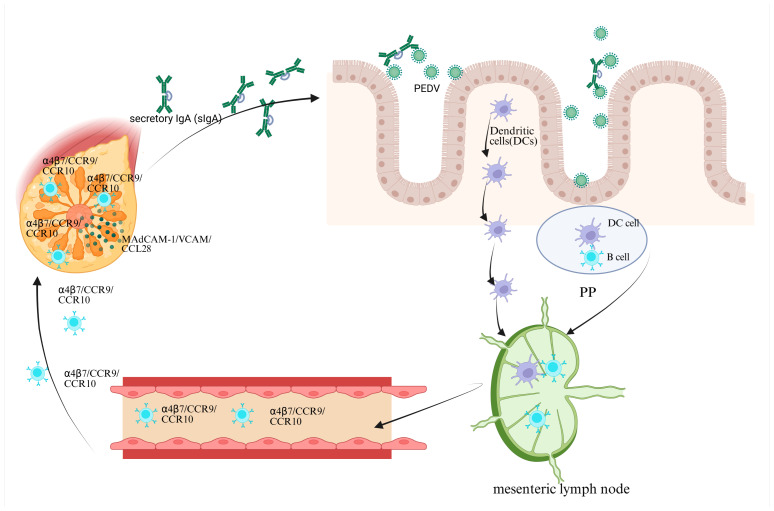
Schematic illustration of the gut–mammary gland–sIgA axis and mucosal lymphocyte trafficking following PEDV infection or oral immunization in sows. PEDV infection or the administration of live oral vaccines initiates antigen recognition by immune cells (e.g., dendritic cells) within Peyer’s patches (PPs) and mesenteric lymph nodes (MLNs). These events induce IgA class switching and imprint mucosal homing phenotypes on activated B cells through upregulation of integrin *α*4*β*7 and the chemokine receptors CCR9 and CCR10. Subsequently, IgA-secreting plasmablasts exit intestinal inductive tissues, enter the circulation, and migrate to mucosal effector sites through interactions between *α*4*β*7 and mucosal addressin cell adhesion molecule-1 (MAdCAM-1), as well as between CCR9/CCR10 and their corresponding ligands, CCL25 and CCL28. During late gestation and lactation, increased expression of adhesion molecules, including MAdCAM-1 and VCAM-1, together with enhanced CCL28-mediated chemotactic signaling in mammary tissues, promotes the selective recruitment and accumulation of intestinally primed IgA-secreting plasmablasts within the mammary gland. Within mammary tissues, these cells differentiate into plasma cells that secrete dimeric IgA. Subsequently, dimeric IgA is transported across mammary epithelial cells via the polymeric immunoglobulin receptor (pIgR) and released into colostrum and milk as sIgA [18,63,64].

The “gut–mammary gland–sIgA axis” refers to the process in which sows are exposed to antigens through the intestinal tract or mucosa surfaces during pregnancy or the perinatal period. This exposure leads to the activation of antigen-specific B cells in gut-associated lymphoid tissue. These B cells express homing receptors (e.g., CCR9/10 and *α*4*β*7), which enable them to migrate to the mammary gland and differentiate into plasma cells that secrete sIgA (Figure 4). The secreted sIgA is subsequently transferred to colostrum or milk, thereby conferring passive mucosal immunity to suckling piglets [13,62].

Although serum IgG levels reflect overall systemic immune responses, their protective role in local defense against intestinal pathogens remains limited. During lactation, neonatal piglets acquire maternal antibodies through ingestion of colostrum and milk. Among these antibodies, colostrum-derived IgG is the primary immunoglobulin during the early postnatal stage [65,66]. Within the first 24–28 h after birth, before intestinal closure, immunoglobulins including IgG, IgM, and sIgA can be absorbed across the intestinal epithelium of neonatal piglets into systemic circulation. This early absorption of IgG provides transient systemic passive immunity in neonatal piglets. However, approximately 48 h after birth, intestinal closure prevents efficient absorption of immunoglobulins across the intestinal epithelium into the bloodstream. During this stage, milk-derived sIgA gradually becomes the dominant immunoglobulin mediating mucosal protection in the intestinal lumen. Unlike systemic IgG, sIgA primarily functions at the mucosal surface by neutralizing viral particles, preventing their attachment to intestinal epithelial cells, and limiting viral replication. Importantly, sIgA persists in milk throughout the lactation period, thereby providing continuous mucosal protection against intestinal pathogens such as PEDV [13,62].

Therefore, sIgA in colostrum and milk is a key factor in the protection of newborn piglets. The levels of sIgA in colostrum and milk determine the degree of passive immunity in suckling piglets [63,64]. Studies indicate that milk sIgA titers are closely associated with piglet survival and clinical protection, whereas serum IgG levels are only weakly correlated with protection [16]. Moreover, sIgA is resistant to proteolytic degradation and can efficiently neutralize PEDV in the intestinal mucosa [13]. Collectively, these findings highlight the role of sIgA in passive immune protection and in the evaluation of vaccine efficacy.

## 5. Current Status and Systemic Limitations of Conventional PEDV Vaccines

Due to the absence of specific antiviral drugs against PEDV, vaccination has become the primary strategy for disease control in swine farms worldwide, particularly in China. Currently, commercial vaccines mainly consist of inactivated or attenuated live vaccines derived from GI strains and a limited number of GII variants [67,68]. Since 2013, highly pathogenic G2 group variants (including G2a, G2b, and G2c) have become epidemiologically dominant. Although existing vaccines can partially alleviate diarrhea severity and increase antibody titers in sow serum and colostrum, their clinical protective effects in piglets are inconsistent and their efficacy in preventing viral transmission remains limited [69]. This highlights the limited adaptability of current vaccination strategies to the rapid mutation and antigenic drift of PEDV, result in antigenic mismatch between vaccine-induced antibodies and circulating strains [70].

### 5.1. Limitation of Inactivated Vaccines and Their Immune Protection

Traditional inactivated vaccines are produced by propagating the virus, followed by inactivation using physical methods (e.g., heat treatment) or chemical (*β*-propiolactone) methods [71]. These vaccines typically consist of whole-virus particles or virus-derived subunit antigens [72]. Although inactivated vaccines are highly safe, easy to produce on a large scale, and capable of inducing a strong systemic IgG response, their antigens are not expressed in host cells. In addition, current commercial vaccines are derived from previously circulating strains. As PEDV continues to evolve, especially through accumulated mutations and new glycosylation patterns in the S1-NTD/COE regions, the antigenicity of currently circulating G2c strains has changed, leading to reduced antigenic matching with vaccine strains.

### 5.2. Efficacy of Attenuated Live Vaccines Accompanied by Biosafety Risks

Compared with inactivated vaccines, attenuated live vaccines can undergo limited replication in the host and more closely mimic the natural infection process of PEDV.

However, attenuated live vaccines have several limitations. First, serial passage remains the primary method for generating attenuated strains. In many cases, when PEDV is sufficiently attenuated in piglets, its replication capacity is also significantly reduced [73]. Second, antigenic variation among PEDV genotypes limits cross-protection. Cross-protection between GI and GII strains is relatively weak, and there are also differences in protective efficacy between G2a and G2b strains [74]. In addition, attenuated live vaccines pose potential biosafety risks, including virulence reversion, within-herd transmission, and recombination with wild-type strains. These issues are particularly important in large-scale farms under high viral pressure. In summary, these findings indicate that the major challenge facing attenuated live PEDV vaccines is maintaining a balance among mucosal immunogenicity, genetic stability, and biosafety in the context of continuous viral evolution.

### 5.3. Advantages and Practical Bottlenecks of Subunit Vaccines

Subunit vaccines usually use the PEDV S protein or its functional domains (e.g., COE, SS2, 2C10, or RBD) as immunogens, along with the addition of adjuvants. They have advantages such as high biosafety, no risk of replication, and strong scalability [75].

To date, most PEDV subunit vaccines are designed on soluble proteins or linear epitopes, making it difficult to reproduce the natural trimeric shape of the viral S protein. Consequently, the immune response often targets a limited set of accessible linear epitopes and fails to induce broadly neutralizing antibodies against conformational epitopes. Furthermore, these vaccines are usually administered via intramuscular or subcutaneous injection, which poorly stimulates the intestinal mucosal immunity and makes it difficult to generate a potent and durable milk IgA response [76]. In the current epidemiological context, where G2/G2c strains predominate and antigenic drift continues to occur, these immunological and structural limitations of subunit vaccines further constrain their effectiveness in practical applications.

Therefore, despite the advantages of subunit vaccines in safety and engineering flexibility, systematic optimization is still required in antigen conformation presentation, mucosal immune induction, and adaptability to rapidly evolving strains.

## 6. Strategies for Broad-Spectrum Antigen Design and Structural Optimization

### 6.1. Structural and Conformational Basis of the S Protein

With the continuous evolution of PEDV strains and the reduced efficacy of conventional vaccines, structure-based antigen design has become an important direction for next-generation vaccine development. The PEDV S protein mainly exists as a trimer, in which the receptor-binding domain (S1-CTD) is usually maintained in a closed (“down”) state. Through hinge-like movements, S1-CTD can shift from the “down” to the “up” state, thereby exposing receptor-binding sites. The “up” conformation exposes key neutralizing epitopes more efficiently and is more readily recognized by neutralizing antibodies [77]. Therefore, the dynamic transition between the “up” and “down” conformations of the prefusion trimer is closely associated with epitope exposure and antibody recognition.

Recent advances in structural biology have enabled high-resolution analyses of coronavirus S proteins, revealing that many neutralization-sensitive epitopes are mainly exposed in the prefusion trimeric conformation. In this state, the S protein maintains a metastable conformation that supports receptor binding while preserving critical conformational epitopes recognized by potent neutralizing antibodies [78]. However, coronavirus S proteins are inherently unstable and may undergo irreversible conformational transitions from the prefusion to the postfusion state during protein translation, purification, or membrane fusion activation [78]. This transition is characterized by substantial refolding of the S2 subunit, leading to the loss or masking of important prefusion-specific epitopes and reduced ability to elicit broadly neutralizing antibodies. Cryo-EM investigations of SARS-CoV-2 spike protein have also shown that most potent neutralizing antibodies selectively target epitopes that are maintained in the prefusion trimer but are absent or structurally changed in the postfusion state [79].

To stabilize the S protein in its prefusion conformation, researchers have developed structure-guided antigen engineering strategies. Double proline substitutions (“2P” mutations) were first introduced into the central helix and HR1 region to restrict the conformational flexibility required for membrane fusion and thereby stabilize the prefusion structure [78,79]. Additional proline substitutions (“6P” mutations) were later introduced, further improving trimer stability, increasing resistance to thermal denaturation and conformational decay, and enhancing retention of the antigenically relevant prefusion state [79]. Compared with unstabilized S proteins, these prefusion-stabilized immunogens exhibit greater structural uniformity, improved antigen presentation, and enhanced induction of neutralizing antibodies.

Moreover, researchers have employed computational modeling and structure-based design to introduce hydrophobic substitutions between the SD1 and S2 subdomains, such as A570L and T572I. These mutations disrupt the closed state, thereby reducing the energetic barrier to conformational opening. As a result, the S protein is more likely to adopt the “RBD-up” conformation and can even assume a rare “2-up” state in which two RBDs are simultaneously exposed. This “forced-opening” design can enhance immunogenicity and elicit stronger neutralizing antibody responses [80].

Prefusion stabilization has now become a major strategy in coronavirus vaccine design and has been widely applied in mRNA-, recombinant protein-, and nanoparticle-based vaccines. Although most mechanistic evidence has been derived from studies of the SARS-CoV-2 S protein [78,79,80], structural studies of the PEDV S protein have revealed similar prefusion metastability and epitope exposure patterns [77]. Therefore, structure-guided prefusion stabilization strategies are likely applicable to PEDV vaccine design and may assist in addressing the ongoing antigenic drift of circulating strains.

Collectively, stabilization of the prefusion S protein is increasingly recognized as an effective strategy for improving coronavirus vaccine immunogenicity and enhancing neutralizing antibody responses.

### 6.2. Strategic Value of the Conserved S2 Region

With the evolution of PEDV and decreasing efficiency of current vaccines, the S2 subunit has been increasingly focused on as a target for broad spectrum immunity. The S1 subunit primarily facilitates receptor binding and is under significant immunological selection pressure, whereas the S2 subunit mediates membrane fusion and is more evolutionarily conserved among PEDV strains. Such conservation provides a fundamental basis for cross-strain immune recognition. Functional investigations have demonstrated that the six-helix bundle produced by HR1-HR2 contacts is important for membrane fusion and can also elicit neutralizing antibodies [81]. These findings indicate that the S2 region is not only essential for viral entry, but also serves as a promising target for antiviral and immunogenic purposes.

Based on these structural and functional characteristics, increasing efforts have focused on incorporating the S2 region into cross-protective or chimeric vaccine designs. For example, replacing or integrating S2 regions from different strains may preserve S-protein immunogenicity while enhancing the breadth of protection, thereby providing new strategies for broad-spectrum PEDV vaccine development [82]. In contrast to the highly variable S1 subunit, S2 is considered an important target for achieving cross-strain protection because of its structural conservation and essential function. Accordingly, it holds substantial value for the rational design of broad-spectrum PEDV vaccines.

### 6.3. Polyvalent Antigen Design

As PEDV continues to diversify into multiple genetic lineages, traditional vaccines based on a single strain or antigen exhibit limited protective efficacy and fail to provide robust cross-protection. Studies have shown that although acid sequences of the S protein are relatively conserved among PEDV strains, their antigenic epitopes differ substantially. For example, an analysis of B-cell epitopes in the vaccine strain CV777 and the epidemic strain SD2014 revealed that, despite over 92% sequence similarity in the S protein, only 25–30% of antigenic epitopes were shared. This finding suggests that antigenic conservation is substantially lower than sequence conservation. Such epitopes difference can alter antibody recognition and contribute to immune evasion and strain replacement [25,83].

Given the complex epidemiological situation of PEDV, traditional single-antigen vaccines provide only limited protective coverage. Therefore, polyvalent and chimeric antigen design has become a major focus in the development of next-generation PEDV vaccines. The primary objective of these strategies is to expand antigenic coverage and enhance cross-protection against diverse strains.

Polyvalent antigen expression strategies have shown considerable promise in achieving broad-spectrum protection against diverse PEDV strains. Several studies have used the S proteins of the G2a and G2b strains to construct bivalent subunit vaccines. In both active and passive immunization models, these vaccines induced IgG and IgA responses against both G2a and G2b strains in piglets and sows. In challenge studies, they alleviated clinical symptoms and reduced pathological lesions in piglets. Although this strategy did not significantly improve complete protection rates, it expanded the breadth of protection and improved clinical outcomes [84]. Therefore, multiepitope and polyvalent antigen design strategies remain promising approaches for the development of broad-spectrum PEDV vaccines. In addition, chimeric antigen design strategies provide new opportunities for the development of broad-spectrum PEDV vaccines. Chimeric vaccines have been generated by replacing the S2 subunit of the G2-type PEDV S protein with the corresponding region from low-pathogenic G1-type PEDV strains. Animal studies demonstrated that this chimeric vaccine induced broadly neutralizing antibodies against both G1 and G2 PEDV strains and conferred potential cross-protection in piglet challenge models [82].

Although optimized antigen design can enhance the quality and breadth of vaccine-induced immune responses, antigen selection alone is unlike to provide sufficient protection against PEDV. To enhance protective immunity, advances in antigen engineering must be complemented by vaccine platforms capable of efficiently delivering antigens and activating the gut–mammary gland–sIgA axis, which is essential for the induction of mucosal lactogenic immunity. Therefore, integrating rational antigen design with platform technologies that enhance mucosal immune induction will be critical for the development of next-generation PEDV vaccines.

## 7. Platform Upgrades: From Systemic Immunity to Mucosal-Targeted Immunity

### 7.1. Nanoparticle Vaccines

Nanoparticle vaccines display the S protein or its key neutralizing epitopes in a multivalent manner on nanoparticle surfaces, enabling high-density and controlled antigen presentation and thereby significantly enhancing immunogenicity [85]. This architecture enhances B-cell receptor cross-linking, promotes robust humoral immune responses, and increases neutralizing antibody titers. In addition, nanoparticle platforms improve antigen stability and facilitate antigen delivery to antigen-presenting cells, thereby further strengthening immune responses.

Recent studies have employed Helicobacter pylori ferritin as an antigen delivery scaffold to develop nanoparticle vaccines that display the PEDV core neutralizing epitope (COE). Compared with monomeric COE vaccines, this platform induces higher levels of IgG, IgA, and neutralizing antibodies in sow serum and colostrum. In piglet challenge studies, 3-day-old piglets born to immunized sows exhibited no clinical symptoms following challenge with virulent PEDV, experienced reduced weight loss, and rapidly cleared virus from fecal swabs and intestinal tissues. These findings indicate that nanoparticle vaccines can effectively induce lactogenic antibody responses in sows and confer passive protection to newborn piglets [86,87].

Nevertheless, despite the encouraging protective efficacy observed in experimental studies, the practical application of nanoparticle vaccines in PEDV vaccination strategies remains a challenge. From an immunological perspective, most studies to date have primarily focused on evaluating systemic antibody responses. However, the ability of nanoparticle vaccines to induce durable intestinal mucosal immunity and sustained milk sIgA responses has not been fully characterized. Optimizing mucosal delivery routes and adjuvant formulations capable of activating the gut–mammary gland–sIgA axis remains a significant challenge, as effective protection against PEDV is largely dependent on lactogenic immunity.

From an antigenic perspective, the breadth of protection provided by current vaccine candidates may be compromised by the ongoing evolution of circulating G2 and G2c strains, as well as alterations in spike glycosylation patterns. Consequently, achieving broader cross-protective immunity may require the incorporation of prefusion-stabilized S proteins, conserved epitopes, or multivalent antigen designs.

Furthermore, nanoparticle vaccine platforms face several technical and translation limitations. Batch consistency and reproducibility may be affected by variations in nanoparticle size, antigen density, and antigen-loading efficiency. Maintaining antigen stability during formulation, storage, and large-scale manufacturing also remains technically challenging. In addition, data regarding the safety of repeated immunizations, long-term immune persistence, and the induction of lactogenic immunity in pregnant sows under commercial farming conditions remains limited. Together with manufacturing complexity and production costs, these challenges may hinder large-scale commercialization. Consequently, future research should focus on optimizing antigen design and enhancing mucosal immune responses, improving manufacturing robustness, evaluating long-term safety and field efficacy, and developing scalable production strategies suitable for commercial swine production systems [88].

### 7.2. Structure-Guided Trimeric Antigen Vaccines

In coronavirus vaccine development, maintaining the native conformation of the S protein is critical for inducing effective neutralizing antibody responses. The PEDV S protein naturally exists as a trimer, whereas many early subunit vaccines expressed only monomeric S1 protein, which failed to recapitulate the native trimetric structure and consequently exhibited reduced immunogenicity. Advances in structural biology have facilitated the development of structure-guided antigen design strategies aimed at stabilizing the native conformation of the S protein. Several studies have employed yeast expression systems to produce trimeric S1 fusion proteins together with FP antigens derived from wild-type PEDV sequences. In this platform, the C-terminal pro-peptide of human type I collagen (Trimer-Tag) was fused to the PEDV antigen, promoting homotrimer formation through disulfide-bond interactions and helping preserve the native structure of the S protein. Immunization studies in mice and sows demonstrated that this vaccine induced robust humoral immune response, characterized by high IgG titers and enhanced mucosal IgA responses. Challenge studies in pigs further demonstrated reduced viral loads and alleviated clinical symptoms [89]. These findings suggest that mimicking the native viral structure can enhance the immunogenicity of PEDV subunit vaccines.

Although trimerization strategies improve structural authenticity, several challenges remain. One major challenge is that post-translational modifications differ among expression systems. For example, glycosylation patterns in yeast and other recombinant expression systems differ from those in mammalian cells, potentially altering epitope exposure on the S protein and affecting antibody recognition and neutralizing activity [51]. Therefore, future studies should employ mammalian expression systems that more closely mimic natural infection conditions or optimize glycosylation pathways to improve antigen authenticity. In addition, future vaccine design should incorporate prefusion trimer stabilization strategies that have been developed for other coronaviruses to further improve neutralizing antibody responses [90,91,92].

### 7.3. Nucleic Acid Vaccines

Nucleic acid vaccines are modern vaccine platforms that enable antigen expression directly within host cells and partially mimic natural viral infection. This category mainly includes DNA and RNA vaccines. Current studies on PEDV DNA vaccines mainly focus on the S protein as the core antigen and aim to induce both humoral and cellular immune responses using eukaryotic expression plasmids. Several studies have developed DNA vaccines encoding the full-length PEDV S protein, the S1 subunit, or porcine IL-18. Mouse immunization experiments showed that pVAX1-(PEDV-S) induced antibody production, lymphocyte proliferation, and IFN-γ secretion. However, the adjuvant effect of IL-18 was limited, suggesting that PEDV DNA vaccines can induce both humoral and cellular immunity to some extent [93]. Subsequent studies compared recombinant plasmids expressing either the S1 subunit or the full-length S protein. The full-length S protein more effectively promoted T-cell proliferation, increased CD4+ and CD8+ T-cell populations, and induced higher levels of antigen-specific and neutralizing antibodies [94].

With increasing understanding of PEDV pathogenesis and immunity, maternal immunity has become an important focus in vaccine development. Consequently, DNA vaccine research has shifted toward optimizing mucosal immunity and delivery strategies to enhance protection in neonatal piglets. One study employed circular polymer nanotubes (cPNTs) as both a delivery system and adjuvant for an oral DNA vaccine encoding the full-length PEDV S protein in pregnant sows. This strategy increased IFN-γ and IL-12 expression, elevated serum IgA levels, and promoted the expansion of CD4+ and CD8+ T cells. Following viral challenge, piglets experienced only minor diarrhea, reduced viral shedding and normal weight gain [95]. These findings suggest that the combination of DNA vaccines with mucosal delivery systems may enhance maternal sIgA responses and provide passive protection to neonatal piglets. DNA vaccines have several general advantages, such as flexible design, good thermal stability and relatively low production cost. However, inefficiencies in cellular uptake and intracellular transport of plasmid DNA often limit the immunogenicity of DNA vaccines, which is generally considered lower than that of mRNA-based platforms [96]. To date, several strategies, including codon optimization, electroporation-assisted delivery, and co-expression with molecular adjuvants, have been employed to improve the efficacy of DNA vaccines [76]. Furthermore, information regarding the long-term protective efficacy and the persistence of lactogenic immunity in pregnant sows under field conditions remains limited.

mRNA vaccines rely on the delivery of synthetic mRNA encoding the target antigen into host cells via specific delivery systems. Once inside the cells, the mRNA is translated into antigenic proteins that are processed and presented to the immune system, inducing both humoral and cellular immune responses [97]. In recent years, mRNA vaccines have emerged as one of the most rapidly advancing vaccine platforms owing to their rapid design, short production cycles and strong immunogenicity. They have demonstrated considerable potential for the prevention of viral infections, such as SARS-CoV-2, influenza, and hepatitis C [98,99,100]. Researchers have developed lipid nanoparticle-encapsulated mRNA vaccines (mRNA-LNPs) encoding the full-length PEDV S protein. Immunizations induced high levels of PEDV-specific IgG and IgA antibodies in the serum and colostrum of vaccinated sows. Following viral challenge, piglets receiving maternally derived antibodies exhibited a low incidence of diarrhea, significantly reduced virus shedding, and relatively mild intestinal lesions [101].

Despite these advantages, mRNA vaccines still face several limitations. mRNA molecules are inherently unstable and generally require stringent cold-chain storage and transportation conditions, thereby limiting large-scale application [102]. Furthermore, extracellular RNases quickly degrade mRNA, necessitating protective delivery systems such as lipid nanoparticles to ensure stability and efficient intracellular delivery [76]. High manufacturing costs also remain a major challenge. Moreover, the long-term safety, persistence of lactogenic immunity, and protective efficacy of repeated immunization in pregnant sows have not been comprehensively evaluated. Therefore, further optimization of formulation stability, field applicability, and delivery systems remains necessary.

To overcome these limitations, several optimization strategies have been proposed. Nucleotide modifications and improved lipid formulations can enhance mRNA stability and protein expression efficiency, thereby improving thermal stability and reducing dependence on cold-chain storage [103]. In addition, the development of RNase-resistant delivery systems may further reduce mRNA degradation and improve delivery efficiency and antigen expression [104].

With continued technological advances, the production costs of mRNA vaccines are expected to decrease, whereas their immunogenicity and accessibility are likely to improve further. Concurrently, next-generation mRNA vaccines that target conserved epitopes or incorporate multivalent antigen designs are expected to broaden cross-protective coverage and enhance protection against diverse viral strains.

### 7.4. Virus-like Particle Vaccines

Virus-like particles (VLPs) consist of one or more viral structural proteins and closely mimic the morphology of native viruses, while lacking a viral genome. Compared with inactivated or live attenuated vaccines, VLPs provide superior safety and immunogenicity, eliminate the risk of reversion to virulence, and induce more robust immune responses [105].

PEDV virions are namely composed of three major structural proteins: S, M, and E. When co-expressed in eukaryotic expression systems, these proteins can self-assemble into PEDV VLPs. Electron microscopy analyses have demonstrated that PEDV VLPs closely resemble native virions in both morphology and structure and exhibit strong immunogenicity in mouse models [106].

Subsequent studies employed multisense retrovirus expression vectors to generate VLPs containing the PEDV S, M, and E proteins. These VLP vaccines induced systemic anti-PEDV S-specific IgG responses and cellular immunity following intramuscular immunization. Moreover, co-administration of PEDV VLPs with CCL25/28 adjuvants significantly enhanced systemic IgG, mucosal IgA, and cellular immune responses while reducing viral shedding and alleviating clinical symptoms in challenge studies [107]. In another study, a B-cell epitope derived from the PEDV S protein (sequence: 748YSNIGCK755) was inserted into the HBcAg capsid to generate a VLP vaccine, which was subsequently evaluated in pregnant sows. This vaccine induced higher levels of neutralizing antibodies in sows and, through maternal immunity, significantly reduced disease severity and improved survival rates in neonatal piglets [108].

Recent studies comparing PEDV VLP vaccines produced in different expression systems have showed that mammalian-derived VLPs elicited superior immune responses. Compared with S subunit vaccines and commercial inactivated vaccines, VLP vaccines induced higher IgG, IgA, and neutralizing antibody titers, as well as more robust lymphocyte proliferation response. Among these platforms, mammalian cell-derived VLP vaccines exhibited the strongest immunogenicity [109]. Collectively, VLP vaccines represent a promising antigen delivery platform for PEDV vaccine development because they mimic the native virion structure and can be combined with strategies that enhance mucosal immunity.

Despite the exceptional safety and immunogenicity of PEDV VLP vaccines, their practical application is still constrained by several challenges. Although numerous studies have demonstrated that PEDV VLP vaccines can induce mucosal IgA responses and confer passive protection to neonatal piglets, the durability of lactogenic immunity and the persistence of sIgA under commercial production conditions remain largely unknown. Given that passive mucosal immunity represents the principal mechanism underlying protection against PEDV, strategies aimed at enhancing activation of the gut–mammary gland–sIgA axis warrant further investigation.

From a platform perspective, VLP vaccines still face substantial challenges in large-scale production, particularly during downstream purification and industrial manufacturing. Current studies have shown that VLP purification is often hindered by low yields, particle aggregation, and residual host-cell contaminants, compromising purification efficiency and product quality [110]. In addition, conventional purification approaches, including density-gradient ultracentrifugation and chromatographic separation, are labor-intensive, costly, and difficult to scale up for industrial production, thereby limiting the widespread application of VLP vaccines [111]. Additionally, batch consistency and immunogenicity may be affected by variations in protein folding, particle assembly, and glycosylation patterns across expression systems.

In addition, the ongoing antigenic evolution of circulating G2 and G2c strains may compromise the breadth of protection conferred by VLP vaccines based on a single spike antigen. Further studies are required to evaluate the duration of immunity, the safety of repeated immunizations, and the efficacy of protective measures in pregnant sows in commercial production conditions. Future efforts should focus on optimizing multivalent antigen design, improving production efficiency, and establishing scalable and cost-effective manufacturing processes to facilitate the commercialization of PEDV VLP vaccines [112].

### 7.5. Viral Vector Vaccines

Viral vector vaccines use genetically engineered viral vectors to deliver antigen-encoding genes into host cells, thereby enabling in vivo antigen expression and inducing both humoral and cellular immune responses without the need for exogenous adjuvants. Compared with conventional subunit vaccines, viral vectors can support sustained antigen expression in host cells, which enhances antigen presentation and promotes long-term immune memory. Given that PEDV primarily infects intestinal epithelial cells, the development of viral vector vaccines capable of eliciting robust mucosal immunity has become a major focus of research.

Adenoviral vectors represent one of the most extensively investigated viral vector platforms in PEDV vaccine research. Adenoviral vectors can moderately activate innate immunity without provoking excessive inflammatory responses such as cytokine storms. This balanced innate immunity facilitates adaptive responses against the transgene product without inducing excessive adverse effects [113]. Adenoviral vectors induce antigen-specific immune responses, including antibody production and activation of cytotoxic T lymphocytes (CTLs), and are capable of eliciting potent CTL responses at mucosal sites [114,115].

Recent studies have developed a recombinant adenovirus type 5 (rAd5)-based vaccine co-expressing the PEDV S1 and N proteins and evaluated its immunogenicity and protective efficacy in mouse and piglet models. In mice, the vaccine induced robust and durable mucosal and systemic immune responses, with S1-specific IgA remaining detectable in mucosal tissues and serum for up to 12 weeks after immunization. In challenge studies, vaccinated piglets exhibited reduced viral shedding, shortened durations of diarrhea, and less severe intestinal lesions [116].

In summary, viral vector vaccines offer several advantages, including efficient antigen expression, simultaneous induction of humoral and cellular immunity, and the ability to stimulate mucosal immune responses. However, several challenges still limit the practical application of viral vector vaccines against PEDV. One of the primary concerns is the potential for pre-existing anti-vector immunity, particularly against adenovirus serotype 5, which may reduce transgene expression and compromise vaccine efficacy. Moreover, repeat administration of the same vector may enhance anti-vector immune responses, thereby limiting the efficacy of homologous booster immunization. From a platform perspective, the incorporation of multiple antigens or complex immunogens may be constrained by the limited payload capacity of viral vectors. Moreover, the technical complexity and high cost associated with large-scale production, purification and quality control pose substantial challenges for industrial manufacturing.

Furthermore, most studies have been conducted in mice or neonatal piglets, leaving significant gaps in knowledge regarding the safety of repeated immunizations, long-term immune persistence, and lactogenic immunity in pregnant sows under field conditions. In addition, the ongoing antigenic evolution of circulating G2 and G2c strains may compromise the breadth of protection provided by current viral vector vaccines expressing a single S antigen. Consequently, future research should focus on optimizing vector design, developing heterologous prime-boost strategies, incorporating conserved or multivalent antigens, and evaluating protective efficacy under commercial swine production condition.

### 7.6. Engineered Bacterial and Oral Vaccines

Driven by the need to enhance mucosal immunity, engineered probiotics have emerged as promising oral vaccine delivery vehicles against PEDV. This strategy employs genetic engineering to express PEDV antigens in generally recognized-as-safe microorganisms, such as lactic acid bacteria, which are administered orally to the intestinal mucosa, where they activate gut-associated lymphoid tissue (GALT) and induce mucosal responses.

*Lactobacillus* species are currently the most widely used bacterial carrier for oral PEDV vaccine development. Lactic acid bacteria can colonize the gastrointestinal tract and possess intrinsic immunostimulatory properties [117]. Moreover, Lactobacillus species can induce sIgA, activate innate immune cells, and modulate T-cell subset balance [118]. Researchers have engineered Lactobacillus to deliver a fusion protein consisting of the PEDV core neutralizing epitope (COE) and a dendritic cell-targeting peptide (DCpep). Upon oral administration, this vaccine induced mucosal sIgA responses and systemic IgG-mediated humoral immunity. In challenge studies, piglets immunized with this engineered *Lactobacillus*-based vaccine reduced intestinal damage and enhanced antiviral protection [119].

Recent advances in antigen delivery strategies have focused on targeting intestinal immune cells to improve vaccine efficacy. Microfold (M) cells and dendritic cells (DCs) play key roles in antigen uptake and immune activation; therefore, targeting these cells can therefore significantly improve vaccine immunogenicity. One study developed an M cell-targeted oral vaccine based on *Lactobacillus casei* expressing the PEDV COE antigen and incorporating the M cell-targeting peptide Co1 as a mucosal adjuvant. In mice, oral immunization induced higher anti-PEDV serum IgG levels and stronger mucosal sIgA responses. Compared with COE-only vaccines, addition of Co1 enhanced splenic lymphocyte proliferation and promoted a Th2-biased anti-PEDV immune response [120].

Further studies have fused both M cell- and dendritic cell-targeting peptides to PEDV antigens to generate dual-targeted oral vaccines. In mouse models, these vaccines induced higher mucosal sIgA levels and stronger PEDV-neutralizing antibody activity. In addition, orally immunized mice exhibited enhanced splenic lymphocyte proliferation and markedly elevated levels of the Th2-associated cytokine IL-4 [121].

More recently, multiple immunostimulatory strategies have been incorporated into engineered bacterial vaccines. For example, one study constructed a recombinant *Lactobacillus paracasei* strain expressing a fusion antigen composed of PEDV S1, immune cell-targeting peptides, and a mucosal adjuvant. Orally immunized pregnant mice exhibited elevated PEDV-specific serum IgG levels and increased sIgA in the intestinal mucus and fecal samples. Moreover, oral immunization enhanced cellular immune responses. Notably, offspring born to immunized dams exhibited higher levels of PEDV-specific sIgA, indicating effective maternal antibody transfer [122].

In summary, engineered bacterial oral vaccines can enhance maternal immunity by improving antigen delivery and promoting mucosal immune responses. Although these vaccines have several advantages, including induction of mucosal immunity, a favorable safety profile, and suitability for mass immunization, they still face several important challenges. Gastric acid, bile salts, and digestive enzymes may reduce the survival of recombinant bacteria, thereby leading to variable antigen delivery efficiency. In addition, heterologous antigen expression is frequently unstable. Furthermore, interindividual differences in intestinal microbiota composition and bacterial colonization efficiency may contribute to inconsistent immune responses among hosts. Repeated oral administration may also lead to variability in vaccine efficacy or the induction of oral immune.

From a translational perspective, the potential risk of horizontal gene transfer and environmental dissemination, together with biosafety and regulatory concerns associated with genetically modified microorganisms, continue to represent major barriers to commercialization. In addition, most studies have been conducted in mice, and information regarding the safety of repeated immunizations, protective efficacy, and long-term lactogenic immunity in pregnant sows under commercial production conditions remains limited. Future research efforts should focus on optimizing antigen expression and secretion systems, integrating targeted delivery strategies with mucosal adjuvants to improve vaccine efficacy and practical applicability, and incorporating microencapsulation or nanoencapsulation technologies to enhance gastrointestinal stability.

### 7.7. Current and Emerging PEDV Vaccine Platforms

Overall, compared with conventional vaccines, emerging PEDV vaccine platforms offer distinct advantages in preserving native antigen conformation, inducing mucosal immunity, and enhancing cross-protective potential. These next-generation platforms place greater emphasis on preserving the native structure of viral antigens and efficiently activating the gut–mammary gland–sIgA axis. The characteristic and recent advances in current PEDV vaccine platforms are summarized in Table 1.

Collectively, these findings indicate that future PEDV vaccine development will increasingly incorporate strategies aimed at enhancing mucosal and lactogenic immunity, together with structure-guided antigen design. Flexible platforms, including mRNA vaccines, nanoparticle-based vaccines, and mucosal delivery systems may provide new opportunities to improve vaccine performance and respond to the ongoing evolution of PEDV variants.

However, despite the promising experimental findings, there are still several challenges that must be addressed before these approaches can be translated into practical field applications. Although numerous studies have been conducted under laboratory conditions, evidence from pregnant sows and commercial production settings remains limited. Furthermore, regulatory approval, production costs, delivery efficiency, manufacturing scalability, and antigen stability remain important factors influencing practical implementation. Therefore, further studies are required to evaluate their long-term efficacy, safety, and field applicability under commercial production conditions.

## 8. Reconstruction of the Immune Evaluation System and Protective Endpoints

Traditional immunological evaluation systems have primarily relied on serum antibody responses. Although IgA and neutralizing antibodies are considered important correlates of protection, numerous studies have reported discrepancies between immunological markers and actual protective outcomes. These discrepancies are increasingly attributed to the mismatch between quantitative antibody measurements and functional antibody activity. Several factors may contribute to these discrepancies. ELISA primarily measures antibody binding rather than virus-neutralizing activity mediated by specific IgA antibodies [16]. In addition, serum IgG levels exhibit a weaker correlation with intestinal protection than milk-derived sIgA levels and neutralizing activity [123]. Variability in maternal antibody transfer and the progressive decline of lactogenic antibodies during lactation may further influence outcomes in neonatal piglets [16,124].

Among the available immunological markers, milk-derived sIgA and neutralizing antibodies are more closely associated with protection than serum IgG alone [64]. Functional immune evaluation should distinguish between antibody abundance and functional activity. Therefore, ELISA-based antibody quantification should be complemented by neutralization assays to better assess vaccine-induced protective immunity and functionally evaluate vaccine efficacy [16].

Ultimately, immunological measurements should be interpreted in conjunction with challenge outcomes. Challenge studies have demonstrated that maternal antibodies can reduce viral shedding and alleviate clinical manifestations of disease. However, the quantity of maternally transferred antibodies alone does not necessarily predict protective efficacy in piglets [124,125]. Viral shedding, diarrhea severity, survival rate, weight gain, and intestinal lesion scores remain critical endpoints for evaluating vaccine efficacy under both experimental and field conditions.

## 9. Challenges and Future Strategies for PEDV Vaccines

### 9.1. Antigenic Evolution and Vaccine Mismatch

Current immunization strategies for the prevention and control of PED remain suboptimal, and these limitations have become increasingly evident with the ongoing emergence of antigenically diverse PEDV variants. Under sustained immune pressure, rapid evolution within the S1 region of the S protein has progressively reduced the antigenic compatibility between classical vaccine strains and currently circulating field isolates. Molecular epidemiological studies have revealed substantial genetic diversity within the PEDV S gene across different geographic regions, and these variations are closely associated with reduced vaccine efficacy and incomplete cross-protection [126]. Collectively, these findings suggest that ongoing antigenic evolution, together with insufficient induction of lactogenic immunity, remains a major obstacle to effective PEDV prevention and control.

### 9.2. Enhancing Mucosal and Lactogenic Immunity

Protective immunity against PEDV mainly depends on the gut–mammary gland–sIgA axis. In addition to antigenic mismatch, current immunization strategies do not adequately align with the immune mechanisms required for effective protection against PEDV infection. Therefore, improving the quality and persistence of lactogenic immunity, rather than simply increasing systemic antibody titers, may represent a key strategy for overcoming the limitations of current PEDV vaccines.

However, most currently available vaccines are administered intramuscularly and predominantly induce systemic IgG responses rather than durable mucosal sIgA immunity. Because neonatal protection relies largely on milk-derived sIgA, this mismatch between vaccine-induced immune responses and the mechanisms underlying protective immunity may contribute to the limited efficacy of conventional PEDV vaccines.

In addition to milk-derived antibodies, susceptibility to PEDV infection may also be influenced by other components of the intestinal mucosal barrier. Recent studies have suggested that age-related changes in mucus composition during the weaning period are associated with reduced susceptibility to PEDV infection in older piglets. These findings suggest that the maturation of the intestinal mucus barrier may complement lactogenic immunity in restricting PEDV infection [127].

### 9.3. Structure-Guided Antigen Optimization

Structure-guided antigen optimization may provide a promising strategy for enhancing cross-protective immunity. In addition to enhancing mucosal immunity, optimization of antigen design remains a key strategy for next-generation PEDV vaccines. Structure-guided approaches targeting domains, such as the S2 subunit, may contribute to broader immune responses and enhanced cross-strain protection [128]. These approaches may also facilitate the development of updated or multivalent vaccine antigens that better match the continuously evolving PEDV population.

Despite accumulating evidence supporting the immunological potential of conserved S2 regions, several challenges must still be overcome before these strategies can be translated into practical vaccines. Further studies are required to more precisely define protective epitopes, evaluate the durability of immune responses, and verify protective efficacy in pregnant sows under commercial production conditions. Addressing these challenges will be essential for advancing the development of PEDV vaccines capable of providing broad and durable protection.

### 9.4. Platform Strategies for Enhancing Mucosal Immunity

Several emerging platforms, including mRNA vaccines, nanoparticle-based vaccines, viral vectors, and oral mucosal delivery systems, may provide opportunities to enhance maternal protection against PEDV and promote mucosal immune responses. The integration of optimized antigens with advanced delivery systems may further facilitate the induction of protective immunity in neonatal piglets.

As PEDV continues to evolve, ongoing genomic surveillance may facilitate periodic antigen updates and help maintain vaccine effectiveness against newly emergent variants [129]. In this context, adaptable vaccine platforms capable of rapid antigen replacement may offer additional advantages in responding to the continuous evolution of PEDV. Although experimental findings are promising, several challenges must still be addressed before these platforms can be translated into practical field applications. To date, most studies have been conducted under laboratory conditions, and evidence from pregnant sows and commercial production setting remains limited. Furthermore, issues related to antigen stability, manufacturing scalability, delivery efficiency, production costs, regulatory approval, and long-term safety require further investigation. Consequently, further studies are required to evaluate their cost-effectiveness, practicality, and durability in commercial production environments.

## 10. Conclusions and Future Perspectives

In conclusion, substantial progress has been made in understanding PEDV antigenic variation and maternal mucosal immunity, leading to the development of several next-generation vaccine platforms and antigen design strategies. These studies have yielded promising results under experimental conditions and have provided valuable insights into enhancing cross-reactive immune responses and inducting lactogenic immunity. Nevertheless, current evidence remains insufficient, and there are several important challenges that still need to be addressed. Most studies have been conducted in mice or under controlled experimental conditions, whereas data regarding the safety of repeated immunizations, protective efficacy, and long-term immune persistence in pregnant sows under commercial production conditions remain limited. Moreover, the development of broadly protective vaccines may be further complicated by the ongoing antigenic evolution of PEDV, particularly among G2 and G2c strains.

Conserved epitopes, multivalent antigen designs, mucosal delivery approaches, and emerging vaccine platforms may provide promising opportunities to enhance lactogenic immunity and broaden antigenic coverage in future vaccine strategies. However, further studies are required to evaluate protective efficacy against genetically diverse PEDV strains before they can be widely implemented in the swine industry.

## Figures and Tables

**Table 1:** **Comparison of current and emerging PEDV vaccine platforms.**

Vaccine Platform	Immunization Principle	Advantages	Limitations	Development Stage
Inactivated Vaccines	Chemically inactivated whole PEDV	High safety; mature production	Weak mucosal immunity; limited cross-protection	Commercialized
Live-Attenuated Vaccines	Attenuated PEDV mimicking natural infection	Strong systemic and mucosal immunity	Reversion risk; safety concerns	Commercialized
Subunit Vaccines	Recombinant spike proteins or epitopes	High safety; defined antigens	Low immunogenicity; weak mucosal response	Experimental/ clinical
DNA vaccine	Plasmid DNA expressing PEDV antigens	Stable; low-cost; rapid design	Low delivery efficiency; weak in vivo response	Mainly preclinical
mRNA vaccine	LNP-mRNA expressing PEDV antigens	Rapid development; strong immune potential	Instability; cold-chain dependence	Early clinical stage
Virus-like particle (VLP) vaccine	Self-assembled viral protein particles	High immunogenicity; safe	Complex production; scale-up difficulty	Preclinical
Viral vector vaccine	Viral vectors expressing PEDV antigens	Strong cellular and humoral immunity	Anti-vector immunity	Under development
Nanoparticle-based vaccine	Nanoparticle-mediated antigen delivery	Enhanced antigen presentation	Manufacturing complexity	Mostly preclinical
Bacterial vector vaccine	Engineered bacteria delivering mucosal antigens	Strong mucosal IgA induction	Safety and stability concerns	Preclinical

## Data Availability

No new data were created or analyzed in this study.
